# Assessment of competencies in the prevention and control of chronic diseases and their influencing factors among health assistants in Bhutan: a cross-sectional study

**DOI:** 10.1186/s12913-022-08747-z

**Published:** 2022-11-10

**Authors:** Tshewang Lhadon, Nithra Kitreerawutiwong

**Affiliations:** 1 Faculty of Public Health, Naresuan University, Phitsanulok Province, 65000 Thailand; 2 Department of Public Health, Ministry of Health, Thimphu, Bhutan

**Keywords:** Competency, Non-communicable disease, Prevention and control, Health assistants

## Abstract

**Background:**

The morbidity and mortality of chronic diseases are increasing worldwide. The literature confirms that the prevention and control of chronic disease necessitates a robust primary health care system with a competent health care workforce. Studies on competencies in the prevention and control of chronic diseases and their determinants among health assistants (HAs) in Bhutan are scarce. This cross-sectional survey aimed to examine the level of competencies and investigate the factors influencing competencies in the prevention and control of chronic diseases among HAs.

**Methods:**

The sample consisted of 330 HAs who were recruited through simple random sampling. A validated and reliable self-administered questionnaire was used to collect data through a web-based Google Form. Data were analysed using descriptive statistics and multiple regression analysis.

**Results:**

The findings showed that the mean summed competency score was 191 (SD = 25.7). Approximately 96% of the participants perceived that they were competent in the prevention and control of chronic diseases. The multiple regression analysis indicated that work environment (*β* = 0.473), sex (*β* = 0.126), location of the health facility (*β* = − 0.114), and organizational support (*β* = 0.117) affected competencies in the prevention and control of chronic diseases by 31.4% with statistical significance (R^2^ = 0.314) (*p* < 0.05).

**Conclusions:**

This study suggested that improving the number of staff and availability of learning resources, considering training for both sexes, especially female primary health care workers, enhancing mentorship and supervision in rural areas, and establishing the recognition and encouragement of primary health care workers are needed.

## Background

Noncommunicable diseases (NCDs) or chronic diseases are the leading cause of death and disability worldwide [1]. They are now responsible for 74% of all global deaths, 77% of which occur in low- and middle-income countries (LMICs) [2]. Bhutan is a low- and middle-income country (LMIC) that encounters chronic diseases, which increased from 43% in 2010 to 51% in 2019 [3]. Seventy-three percent of deaths and 18% of the projected premature mortality in 2019 were from NCDs [4]. In addition, 80% of the older adults in Bhutan had at least one chronic health condition, and 50% of them had at least two [5]. Lifestyle changes, population ageing, globalization and urbanization are the predominant key drivers of the increase of chronic diseases [6]. Consistent with previous study [7] found that the prevalence of NCD modifiable risk factors such as overweight or obesity and hypertension was high in Bhutan.

The World Health Organization (WHO) Global Status Report on Non-Communicable Diseases 2010 highlighted the importance of strengthening the health system with emphasis on primary health care as the first point of care and partnership between health service providers as the key to success. The attributes of primary health care, as characterized by accessibility, care coordination, continuity of care and comprehensive services, have been able to reduce the mortality rate [8]. In line with the literature, policies and interventions aimed at the management of chronic diseases through primary health care are cost effective and affordable, and enhance equitable distribution of health services [9–12].

In Bhutan, primary health care services are coordinated with secondary care and tertiary care through a referral system. Primary health care services are mainly provided by health assistants (HAs) who are nonphysician frontline health care workers [13]. Chronic disease services require comprehensive, coordinated, and continued services to prevent diseases and their complications and control symptoms [10, 12, 14]. The health system traditionally designed to provide acute episodic care is increasingly known to be inadequate for addressing the long- term health problems of the rising epidemic of chronic diseases [2, 10, 15, 16]. The paradigm shifts disease management from acute episodic care to long-term, proactive and patient-centred care to respond to chronic diseases [17]. Building the competency of health care providers is important to change the service delivery model. Yang et al. ([18] states that NCD management approaches require health care practitioners to be appropriately competent. Furthermore, Mahipala [19] urged that a competent health workforce, essential medicines and technologies, and information systems are key elements for primary health care systems to respond effectively to chronic diseases.

Competency for chronic disease care is defined as a combination of measurable knowledge, skills, abilities, and individual traits that contribute to the prevention, control and provision of care of chronic conditions [14, 17]. The World Health Organization [17] identified five core competencies that the health care workforce needs to prevent, control and provide care for chronic diseases: 1) patient-centred care, 2) partnering, 3) quality improvement, 4) information and communication technology and 5) a public health perspective. WHO suggested that these identified competencies need to prepare the twenty-first century global health care workforce to respond caring for patients with chronic diseases and conditions. Previous studies illustrate that factors such as sociodemographic factors, related training, organizational support, and work environment affect the competency of the health workforce [20–24].

Most of the previous studies examining the factors influencing competencies in the prevention and control of chronic diseases were conducted among nurses in long-term care facilities [20], family physicians [25], and health care leaders in primary care facilities and district health systems [26, 27]. There is a paucity of empirical data concerning the factors influencing competencies in the prevention and control of chronic diseases among HAs, who are major frontline workers in Bhutan. In addition, Bhutan implemented The Service with Care and Compassion Initiative (SCCI) across the country, which emphasizes that HAs in local primary health centres request all levels of hospitals to deliver medication for patients. There were several challenges in the effective implementation of the initiative, including standardizing and designating kits for health workers visiting home-care patients, streamlining the vertical reporting of data, building capacity and a virtual learning environment, and mobilizing and integrating resources into the primary health care approach [28]. In contrast, academics recommend that providing competent human resources and promoting good communication and information systems are critical to improving patient safety in the Bhutanese health care system [29]. Therefore, this study aimed to determine the level of competencies and investigate the predictive factors that affect competencies in the prevention and control of chronic diseases among primary health care workers in Bhutan to design a training program for primary health care workers to increase their competencies for delivering effective chronic care prevention and control for people and communities.

## Methods

### Study design and sites

A cross-sectional study was conducted in health facilities across all 20 districts in Bhutan. This study emphasized the competency of HAs, who are the first point-of-care for the population. All health facilities, including referral hospitals in three regions, district hospitals in every district head quarter, 10 bedded hospitals, primary health centres (PHCs) and sub-posts at the community level across Bhutan, were the unit of analysis. Due to tertiary care and secondary care can provide care given to complex specialist care, and encompass the primary care for the population [30].

### Study population and sample size

The population included 616 HAs. The sample size was calculated using the mean estimation formula with a margin of error (*d*) of 10% (0.044) [26] and a 95% confidence interval (*Z* = 1.96), giving a sample size of 236.84. The desirable of response rate via online survey was 60% therefore, a nonresponse rate of 40% was calculated [31]; thus, the required sample size (*n*) was calculated to be 331.57. The number was rounded up, and the total number as calculated to be 340. The sample from each district was selected using simple random sampling by Statistics Package for Social Sciences (SPSS) computer software after proportional allocation for each district. The information of all HAs including name, email and workplace was derived from the health administrative data, human resource division, Ministry of Health, Bhutan. The principal investigator individually contact HAs by email according to the random number. The inclusion criteria were HAs who had worked in a health care facility for at least 1 year, who were aged 20 years and above and who volunteered to participate in the study. The exclusion criteria were HAs who planned to retire in 6 months and those on leave, such as extraordinary leave and long-term study leave.

### Data collection

The study was approved by the Naresuan University Research Ethics Board of Health (Ref. No. REBH/PO/2022/022). Administrative clearance was sought from the Ministry of Health, Bhutan (MoH/PPD/ADM.CL/9/2022/014). The data for this study were collected using a self-administered questionnaire. The questionnaire was disseminated to the participants using a Google form sent via email. The district health officers (DHO) are the headed of PHCs was the coordinator to facilitate of the data collection process. The online Google Form Survey started with a section that informed the respondents about the purpose of this study, the instructions, the contribution of this study, the voluntary participation and the confidentiality of their responses. The period of data collection was set for 3 weeks. Reminders were sent 1 week after and 1 week before the due date. One week after the second reminder, the due date was extended for follow-up of the late responders; however, no responses were received in that week. The process of data collection was completed within 4 weeks, from 23rd June to 23rd August 2022. Three hundred thirty participants responded to the survey, resulting in a 97% response rate. The data were collected electronically using the principal investigator’s Google Drive. Later, the Google Drive file was downloaded as an excel file.

### Instrument

The questionnaire consisted of 4 sections. Section 1 was designed to obtain data on sociodemographic variables and consisted of 11 items. Section 2 collected data on the work environment and consisted of 10 items adapted from the health care provider work index (HPWI) by McAuliffe et al. [32], including adequate resources, working relationship and control over practices/ autonomy. Section 3 collected data on organizational support and comprised 7 items developed from the literature: incentives, recognition and reward, training opportunities, mentoring, coaching, supportive supervision, and career development. Section 4 assessed the participants’ competencies in the prevention and control of chronic diseases and consisted of the following 5 domains with 48 items: 1) the patient-centred care, 2) partnering, 3) quality improvement, 4) information and communication technology and 5) public health perspective domains adapted from the WHO concept [17] and the 2019 competency-based framework for HAs in Bhutan [33]. The scale format of Section 2–3 of the questionnaire was measured according to a 5-point Likert scale ranging from 1 (strongly disagree) to 5 (strongly agree). For Section 4, the items were scored on a 5-point Likert scale ranging from 1 (lowest performing) to 5 (highest performing). A higher score indicated better results. The content validity was examined by 5 experts, and the validity analysis for the item-content validity index (I-CVI) of the scale was 0.90 [34]. The pilot test was conducted for Section 2–4 of the questionnaire, and the Cronbach’s alpha for each scale, including the work environment, organizational support and competency scales, was 0.90, 0.90 and 0.95, respectively [35].

This study minimized method bias by dividing the questionnaire into 4 sections; therefore, the respondents were required to pause and carefully read instructions. The wording of the scale items was improved to eliminate ambiguity. The instruction on the cover page of the questionnaire was designed to enhance the tendency to respond in a socially desirable manner [36].

### Statistical analysis

The data analysis was performed using the Statistical Package for Social Science (SPSS) version 20.0 software package. Descriptive statistics were employed for sociodemographic factors, work environment, organizational support and competencies. Best JW and Kahn JV [37]. indicated that Likert scale is no basis for belief that the five positions are equally spaces. Therefore, the score of ≥3 from 5 format interpret to be the favourableness point of view. The interpretation cut-off point for work environment was 30. Therefore, a score < 30 indicated an unfavourable work environment, and a score > 30 indicated a favourable work environment. The interpretation cut-off point for organizational support was 21. Therefore, a score < 21 indicated limited organizational support, and a score > 21 indicated adequate organizational support. The interpretation cut-off point for competencies was 150. Therefore, a score < 150 indicated the need for improvement, and a score > 150 indicated competency.

The dependent variable was competency in the prevention and control of chronic diseases, which was measured on a continuous scale. For the independent variables, the continuous variables consisted of age, years in the current facility, work environment and organizational support, and the categorical variables were grouped into the dummy variable of sex, education level, location of the health facility, years of work experience, and work satisfaction. Prior to the main outcome analysis, the assumptions of multiple regression analysis were checked. For normality, a normal P-P plot showed that the residuals (errors) were approximately normally distributed. Multicollinearity was assessed as the tolerance/ variance inflation factor (VIF), which ranged from 0.654–0.980 and 1.02–1.528, respectively. The independence of the observations was assessed using the Durbin-Watson statistic, yielding a value of 1.87. The correlation coefficients of the variables were 0.002–0.681, indicating that there was no multicollinearity between the variables (considering *r* < 0.80) [38]. The assumptions were met for all conducted analyses. Therefore, multiple regression analysis (MRA) was performed to test the hypothesis to investigate the predictive factors of the competencies of the primary health care providers. The significance level was set at *p* < 0.05.

## Results

Overall, data from 330 study participants were analysed in this study. The majority of the study participants (53%) were male, with a mean age of 38.51 ± 9.01 (range 24–57) years. Approximately 87% were married, and 87% had a certificate level of education in community health. Most of the participants were from PHCs (61%), followed by district hospitals (15%), bedded hospitals (12%), sub-posts (7%), referral hospitals (3%) and THCs (2%). Most of the respondents (78%) worked in rural areas. A total of 36% of them reported having more than 20 years of work experience. More than half (58%) of them had been working in the current health facility for 1–5 years. When asked about their experiences on trainings for NCDs, 70.3% reported that they have been trained. The majority of those trained received district-level training (78.4%). Approximately 86.4% of the participants reported being satisfied and very satisfied with their work (Table 1).

**Table 1:** Demographics characteristics of the samples (*n* = 330)

Characteristics	N	Percent (%)
**Age (Year)**		
20–30	80	24
31–40	109	33
41–50	103	31
51–60	38	12
Mean = 38.51, SD = 9.01, Min = 24, Max = 57		
**Sex**		
Male	174	53
Female	156	47
**Marital status**		
Married	286	87
Single (Divorced, separated & widowed)	16	5
Never married	25	8
**Education**		
Certificate	258	78
Diploma	52	16
Bachelor’s degree	20	6
**Type of workplace**		
Referral hospital	11	3
District hospital	48	15
10 Bedded hospitals	38	12
Primary health Center (PHC)	202	61
Thromdey Health Center (THC)	7	2
Sub-post	24	7
**Location of health facility**		
Rural	256	78
Urban	74	22
**Years of work experience**		
1–5 years	58	18
6–10 years	59	18
11–20 years	96	28
> 20 years	117	36
**Years in current job of this facility**		
1–5 years	191	58
6–11 years	93	28
> 11 years	46	14
**Experience on NCD training**		
Ever	231	70.0
Never	99	30.0
Mode of trainings* (*n* **= 231)**		
District level training	181	78.4
Training of trainer	24	10.4
Online course	13	5.6
Multiple method	13	5.6
**Work satisfaction**		
Very unsatisfied and unsatisfied	2	0.6
Neutral	43	13
Very Satisfied and satisfied	285	86.4

* Number decreased due to the response of participants experienced in training. The participants who received more than one approach in training response in multiple method

Table 2 depicts the descriptive statistics of the work environment and organizational support. The scores of the work environment ranged from 10 to 50. Most of the participants felt that their work environment was favourable (79%). The summed mean score for the work environment was 36.28 (SD = 6.43), with the highest score indicating a favourable work environment. In addition, the score for organizational support ranged from 7 to 35. The majority of the participants (77%) reported that their organizational support was limited. The summed mean score for organizational support was 23.19 (SD = 5.00). Summed mean scores of 22 or more indicated adequate organizational support.

**Table 2:** The level of work environment and organizational support. (*n* = 330)

Domain	N	Percent
**Work environment**		
Favorable work environment (31–50)	261	79
Unfavorable work environment (10–30)	69	21
Mean = 36.29, SD. = 6.43, Min = 12, Max = 50		
**Organizational support**		
Adequate support (22–35)	77	23
Limited support (7–21)	253	77
Mean = 23.19, SD. = 5, Min = 7, Max = 35		

The dependent variable comprised five domains, including the patient-centred care, partnering, quality improvement, information and communication technology, and public health perspective domains, as presented in Table 3. The mean summed score of competency was 191 (SD = 25.7), with a maximum score of 240 and a minimum score of 94. Among all the competencies, the highest proportion of participants felt that they were competent in quality improvement (93.3%), with a mean score of 46.25 (SD = 6.51). Approximately 96% of the participants believed that they were competent in carrying out the prevention and control of chronic diseases, and only 4% fell into the need for improvement category.

**Table 3:** The level of the competencies across all five domains (*n* = 330)

Domain	Level of competence				Mean	SD.	Min, Max
	Competence		Need improvement				
	n	%	n	%			
Patient center care	305	92.4	25	7.6	46.25	5.05	21–55
Partnering	305	92.4	25	7.6	46.25	6.51	22–55
Quality improvement	308	93.3	22	6.7	28.9	4.22	12–35
Information and communication technology	282	85.5	48	14.5	41.42	5.21	12–40
Public health perspectives	283	85.8	47	14.2	30.32	5.21	12–40
Overall competencies	316	96	14	4	191	25.7	94–240

For the different domains of competency, patient-centred and partnering competencies were measured by 11 items, each with a total score ranging from 11 to 55. The analysis showed that 92.4% of the participants felt that they were competent. The summed mean score for both the patient-centred care and partnering competencies was 46.25 (SD = 6.05). A total of 93.3% of the participants believed that they were competent in the domain of quality improvement, with a mean score of 28.9 (SD = 4.22). A total of 85.8% of the participants perceived that they were competent in the public health perspective domain. In the domain of information and communication technology, 88.5% of the participants believed they were competent, with a mean score of 41.42 (SD = 5.21). Among all the competency domains, the quality improvement domain (93.3%) showed the highest proportion of competent primary health care workers, and the lowest proportion of competent participants was seen in the information and communication technology domain (85.5%).

The results of the multiple regression analysis with the study of competencies in the prevention and control of chronic diseases as the dependent variable are presented in Table 4. These explanatory variables included work environment (*β* = 0.473), sex (*β* = 0.126), location of the health facility (*β* = − 0.114), and organizational support (*β* = 0.117). These variables predict the competencies in the prevention and control of chronic diseases by 31.4%, with statistical significance (*R*^2^ = 0.314) (*p* < 0.05). According to the results, the predictive equation could be constructed in the Unstandardized Score and Standardized Score by Stepwise technique as follows: The Predictive Equation in the Unstandardized score: Y (competency) = 126.978 + 1.891 (work environment) + 6.456 (sex) - 9.010 (location of the health facility) + 0.600 (organizational support) and The Predictive Equation in the Standardized score: Z (competency) = 0.473 (work environment) + 0.126 (sex) - 0.114 (location of the health facility) + 0.117 (organizational support).

**Table 4:** Multiple regression analysis of the factors predicting the competencies in the prevention and control of chronic diseases among health assistants (*n* = 330)

Variable	B	SEb	β	t	***p***-value
Constant	126.978	8.693		14.607	< 0.001
Work environment	1.891	0.229	0.473	8.269	< 0.001
Sex	6.456	2.425	0.126	2.662	0.008
Location of health facility	−9.010	2.856	−0.114	−2.455	0.015
Organization support	0.600	0.292	0.117	2.054	0.041

*R* = 0.560, *R*^2^ = 0.314, Adjusted *R*^2^ = 0.306, *F* = 4.221, *df* = 1, *p* = 0.041

Variable values: Work environment (Favorable = 1, Unfavorable = 0), Sex (Male = 1, Female = 0); Location of health facility (rural = 1, urban = 0); Organization support (Favorable = 1, Unfavorable = 0)

The correlation coefficients of work environment and organizational support were 0.473and 0.117, respectively, indicating a positive relationship between these variables and competency. This finding indicated that for every one unit increase in the work environment and organizational support variables, competency increased by 0.473 and 0.117, respectively. The variable regression coefficient for sex was 0.126, indicating positive relationships between sex and competency. This indicates that when all other variables remained constant, being male (male = 1 as the reference group) increased competency by 0.126 compared to being female. The variable regression coefficient of the location of the health facility was − 0.114, indicating a negative relationship between the location of the health facility and competency. The result indicates that when all the variables remained constant, the health facility being in a rural area (rural = 1 as the reference group) decreased competency by 0.114 compared to being in an urban area.

## Discussion

According to this study, overall competencies in the prevention and control of chronic diseases were reported by 96% of the respondents (Mean = 191, SD. = 25.7). This situation can be explained by the fact that the Ministry of Health in Bhutan implemented the WHO package of essential noncommunicable (PEN) disease interventions for primary health care (WHO PEN package) by integrating the management of chronic diseases such as diabetes and hypertension into primary health care in 2009 and evaluated the results of a 3-month performance assessment in 2012 that improved the risk of modifying factors and was then rolled out nationwide beginning in 2015 [13, 39]. The Ministry of Health [33] trained health care workers who responded to the WHO PEN package by providing a training program for HAs by health educators that emphasized the public health aspect for the prevention of diseases and promotion of health in communities [3, 33]. The evidence of this study found that 70.3% of the sample had experienced chronic disease training and reported being competent in the prevention and control of chronic diseases. The concept of the competencies in the prevention and control of chronic diseases in this study was consistent with a previous study in the community partnership dimension [21]. In line with previous study [40], indicated that community-based organizations recognize that partnerships are desirable mechanisms for service improvement in chronic disease prevention. To facilitate this result, competency training and refresher training in the prevention and control of NCDs in Bhutan is required for all HAs.

The results also showed that work environment, sex, location of the health facility (rural or urban areas), and organizational support were significant factors that influenced competencies in the prevention and control of chronic diseases among HAs. The work environment was found to be a predictor of competencies in the prevention and control of chronic diseases. HAs who perceived a favourable work environment believed they were competent in the prevention and control of chronic diseases. Other studies revealed that the work environment is relevant to nursing competency [23, 24, 41]. Furthermore, an integrative review found that the work environment is correlated with nursing competency [22]. The work environment in this study was measured by the adequacy of human resources to share the workload, access to learning resources, the availability of drugs and equipment, relationships, and autonomy of practice. The current study showed a moderate score for access to resources such as medicines and equipment for managing NCDs. This is consistent with the PEN clinical audit that reported that 30% of the health facilities in Bhutan experience intermittent stockouts of NCD medicines, have inadequate laboratory reagents and test kits and have limited treatment guidelines [13]. Furthermore, the work relationship item received the highest score in this study, which is compatible with a previous study among physicians in Bhutan [42]. This study suggests that the work environment has the opportunity for improvement in the context of the number of staff and the availability of learning resources.

With regard to sex, being a male was positively and significantly associated with competencies in the prevention and control of chronic disease in this study. This finding is consistent with a systematic review for the association of the factor with nurse and midwife competencies, which reported that males were more competent than their female counterparts [24]. Similarly, a study in Ethiopia [43] noted that males were likely to have better knowledge than females. However, previous studies assessing factors influencing competency among nursing assistants in long-term care facilities in Taiwan [20] and competency among health staff engaged in the prevention and control of chronic diseases in Fiji [21] showed no significant association between sex and competency. In Bhutan, this could be because most of the males who work in primary care facilities work as primary health care managers [26] in addition to clinical positions, and they have more opportunities to attend training, while females mostly handle clinical jobs in health centres. This study suggests promoting opportunities for professional development and sustained encouragement and support for female health care workers.

The location of the health facility (rural or urban areas) was significantly associated with the competency of HAs in Bhutan. HAs who worked in rural areas were negatively associated with competency. This situation explained that in Bhutan, all the HAs were trained to perform the same job responsibilities, however, HAs in rural and urban health care facilities are slightly different in practice. HAs in rural areas need to manage multifaceted demands for various services, such as preventive, promotive, treatment, rehabilitative and administration work, in remote PHCs. HAs working in urban hospitals are engaged in a smaller range of services and have easy access to varied professional consultations. Moreover, working in urban areas requires one to be more competent to deal with more literate and informed urban dwellers [44]. In line with the study of Herberholz C, Phuntshob S [45] reported that there were significant differences in utilization among rural and urban areas in Bhutan. Moreover, with increased access to internet services, health information is one click away for literate individuals. Although health care providers in rural areas are fortunate to have more opportunities for professional development programs such as trainings, workshops, and meetings, being in rural areas was shown to be a negative influencer of competency. Enhanced mentoring and handholding support by supervisors for rural health care workers is recommended to align rural and urban health care workers.

Finally, with regard to organizational support, the current study demonstrated that organizational support positively influences the competency of HAs in the prevention and control of chronic diseases. The result was consonant with a study of primary health care managerial competency [46] reported that intrinsic motivators such as achievement, reward and recognition, responsibility, and advancement and personal growth are positively associated with competency. The components of intrinsic motivators were analogous to the organizational support items in this study. In the current study, it was revealed that most of the participants did not agree that reward and recognition was awarded fairly and adequately (2.92 and 2.90, respectively), with 77% of respondents perceiving that there was limited organizational support in this research. Therefore, recognition and encouragement for the best performers and handholding for poor performers by health care managers and supervisors may help increase competency in HAs.

This study has the limitation of method variance using self-report questionnaire constraints and the responses provided are at risk of being invalid. However, the researcher minimized the error of method bias by maintaining the anonymity of the participants, and the questionnaire did not include personal identifiers. Since this study was conducted among HAs, the results cannot be generalized to other types of health care providers; nevertheless, this study can be generalized to HAs in Bhutan. Beyond addressing the limitations, this study provides the opportunity for future research. The mixed methods design was recommended to triangulate or explain competency. In addition, training programs to enhance competencies in the prevention and control of chronic diseases need to be designed for and tailored to HAs.

## Conclusions

The competencies of this study were investigate based on concept of WHO regarding the competencies for caring for patients with chronic conditions. To our knowledge, this is the first study to examine the competencies in prevention and control and the related factors in Bhutan. The implication of this study should be considered to improve competencies in the prevention and control of chronic diseases among HAs. First, because this study reported that 96% of the respondents were competent, there is room for improvement to sustain their ability such as updating the training program in prevention and control of chronic disease. Second, the work environment has the opportunity for improvement in the context of the number of staff and the availability of learning resources. Third, professional development needs to be supported for both sexes, especially female health care workers. Fourth, enhanced mentoring and handholding support of rural health care workers by supervisors is recommended to align rural and urban health care workers. Finally, recognition and encouragement for the best performers and handholding for poor performers by health care managers and supervisors may help increase competency in HAs.

## Data Availability

The data of this study are available from the corresponding author upon reasonable request due to the protection under the terms of the Naresuan University Ethical Committee for dissemination.

## Abbreviations

HAs: health assistants
SD: Standard deviation
NCDs: Noncommunicable diseases
LMICs: low- and middle-income countries
WHO: World Health Organization
SCCI: The Service with Care and Compassion Initiative
PHCs: Primary Health Centres
SPSS: Statistics Package for Social Sciences
DHOs: District Health Officers
I-CVI: Item-Content Validity Index
VIF: Variance Inflation Factor
MRA: Multiple Regression Analysis
WHO PEN package: The WHO package of essential noncommunicable (PEN) disease interventions for primary health care
